# ^18^F-EF5 PET Is Predictive of Response to Fractionated Radiotherapy in Preclinical Tumor Models

**DOI:** 10.1371/journal.pone.0139425

**Published:** 2015-10-02

**Authors:** Rehan Ali, Sandeep Apte, Marta Vilalta, Murugesan Subbarayan, Zheng Miao, Frederick T. Chin, Edward E. Graves

**Affiliations:** 1 Department of Radiation Oncology, Stanford University, Stanford, CA, United States of America; 2 Department of Radiology, Stanford University, Stanford, CA, United States of America; University of California Davis, UNITED STATES

## Abstract

We evaluated the relationship between pre-treatment positron emission tomography (PET) using the hypoxic tracer ^18^F-[2-(2-nitro-1-H-imidazol-1-yl)-N-(2,2,3,3,3- pentafluoropropyl) acetamide] (^18^F-EF5) and the response of preclinical tumor models to a range of fractionated radiotherapies. Subcutaneous HT29, A549 and RKO tumors grown in nude mice were imaged using ^18^F-EF5 positron emission tomography (PET) in order to characterize the extent and heterogeneity of hypoxia in these systems. Based on these results, 80 A549 tumors were subsequently grown and imaged using ^18^F-EF5 PET, and then treated with one, two, or four fraction radiation treatments to a total dose of 10–40 Gy. Response was monitored by serial caliper measurements of tumor volume. Longitudinal post-treatment ^18^F-EF5 PET imaging was performed on a subset of tumors. Terminal histologic analysis was performed to validate ^18^F-EF5 PET measures of hypoxia. EF5-positive tumors responded more poorly to low dose single fraction irradiation relative to EF5-negative tumors, however both groups responded similarly to larger single fraction doses. Irradiated tumors exhibited reduced ^18^F-EF5 uptake one month after treatment compared to control tumors. These findings indicate that pre- treatment ^18^F-EF5 PET can predict the response of tumors to single fraction radiation treatment. However, increasing the number of fractions delivered abrogates the difference in response between tumors with high and low EF5 uptake pre-treatment, in agreement with traditional radiobiology.

## Introduction

Many solid tumors contain regions of low oxygenation, or hypoxia, due to spatially and temporally inadequate delivery of oxygen by the blood vessels within the tumor mass. The presence of hypoxic regions in solid tumors causes increased resistance to chemotherapy and radiation treatment [1] and is associated with poor clinical prognosis [2]. Radiation resistance in hypoxic cells occurs primarily by limiting oxygen fixation, the process by which radiation-induced DNA damage caused by free radicals is exacerbated by the oxygen-mediated formation of genotoxic DNA adducts. In the absence of oxygen, radiation-induced hydroxyl radicals are extinguished before they can interact with DNA [3]. Historically, the treatment-limiting effects of hypoxia have been mitigated through the use of fractionated radiotherapy. By delivering radiation as a series of temporally-separated doses, normoxic cells are killed while surviving radioresistant hypoxic cells become sensitized through post-treatment reoxygenation [4], facilitating their killing by the next radiation fraction.

Positron Emission Tomography (PET) is a nuclear medicine imaging technique that has been applied over the last 15 years toward the non-invasive, *in vivo* determination of oxygen status. A positron-emitting molecule is injected into the subject and undergoes irreversible reduction-mediated accumulation in hypoxic cells. A variety of PET radiotracers sensitive to hypoxia have been produced, many of which incorporate a 2-nitroimidazole functional group prominent in immunohistochemical hypoxia probes as well as in radiosensitizers. One such compound, ^18^F-[2-(2-nitro-1-H-imidazol-1-yl)-N-(2,2,3,3,3-pentafluoropropyl) acetamide] (^18^F-EF5), has been validated as both an *in vivo* PET imaging agent for hypoxia [5], and as a histological marker for hypoxia in its non-radioactive form [6,7]. Fluorescent antibodies are readily available for immunohistochemical staining of EF5, thus permitting microscopic level verification of hypoxia to be performed alongside ^18^F-EF5 PET imaging using the same agent, albeit at millimolar concentrations that are much higher than the trace picomolar doses used for PET imaging [8].

Previous preclinical studies have shown that pre-treatment hypoxia PET imaging using the radiotracer ^18^F-azomycin arabinoside (^18^F-FAZA) can predict the response of the tumor to single-dose radiation [9] or multiple fractions accompanied by concomitant tirapazamine therapy [10], and that ^18^F-EF5 microPET can predict the response to SN30000 combined with single-dose radiation [11]. To date however, there is no systematic study that evaluates the ability of hypoxia PET to predict the response of tumors to a broad range of fractionated treatments. In this study, we investigated whether pre-treatment ^18^F-EF5 microPET can predict the response of a subcutaneous tumor model to single and multiple fraction radiation treatments. Initially the distributions of hypoxia encountered in several subcutaneous tumor models were characterized in order to identify one that exhibited a range of levels of hypoxia suitable for assessing the influence of pre- treatment imaging signals on treatment response. A large panel of subcutaneous tumors was then imaged with ^18^F-EF5 microPET and treated with a variety of radiotherapy regimens, after which tumor treatment responses were compared with the pre-treatment levels of ^18^F-EF5 uptake. A spectrum of radiation doses and fractionations were utilized in order to comparatively assess the ability of pre-treatment ^18^F-EF5 imaging to predict outcome in each of these treatment regimes. In addition, tumors were imaged longitudinally with post-treatment ^18^F-EF5 microPET in order to investigate the effect of radiation on ^18^F-EF5 uptake. Our results demonstrate distinct tumoral responses to single and multiple dose treatments depending on their pre-treatment ^18^F-EF5 tumor uptake, and suggest that the response of subcutaneous A549 tumors to radiation can be predicted based on the pre-treatment ^18^F-EF5 uptake. Longitudinal post-treatment imaging shows a decrease in ^18^F-EF5 uptake in irradiated tumors and suggests that the observed radiation responses are due in part to reoxygenation.

## Materials and Methods

### Animal models

All animal experiments in this study were approved by the Administrative Panel on Laboratory Animal Care (APLAC) at Stanford University (protocol 17186). All animal procedures described below were conducted under isoflurane anesthesia, and animals were humanely euthanized at the end of the study period via carbon dioxide inhalation. Human HT29 colorectal adenocarcinoma cells, A549 lung carcinoma cells, and RKO colon carcinoma cells obtained from the American Type Culture Collection (ATCC, Manassas, VA) were grown subcutaneously in 8 week old male *nu/nu* nude mice (Charles River, Wilmington, MS). To produce bilateral subcutaneous tumors, 106 tumor cells were injected beneath the skin on each shoulder of the mouse. Animal health was monitored daily, while tumor growth was measured weekly with sliding scale calipers, and tumors were grown for 4 to 6 weeks to a diameter of approximately 7 mm prior to imaging. Animals were monitored for signs morbidity including weight loss, crunched position, and passive behavior, and were humanely euthanized if any of these were noted. Animals were also humanely euthanized once their tumors reached a maximum volume of 1.5 cc.

### Radiochemistry

The EF5 precursor 2-(2-nitro-1H-imidazol-1-yl)-N-(2,3,3-trifluoroallyl)acetamide as well as cold EF5 standard were provided by Varian Bioscience (Palo Alto, CA). Trifluoroacetic acid (TFA) was purchased from Sigma-Aldrich (St. Louis, MO). HPLC- grade solvents were purchased from EMD Chemicals (Philadelphia, PA). Sterile filters (Millex-GS 0.22μM) were purchased from Millipore (Billerica, MA). ^18^F-EF5 was synthesized with a TracerLab FX-FE automated synthesis module (GE Health Care). ^18^F2 gas was produced on a PETrace cyclotron (GE Health Care) through a 20Ne(d, μ)^18^F reaction using a target that was initially filled with a 1% F2/Ne mixture at 50 psi, then was filled to 150 psi with pure Ne, and bombarded by a 40 μA deuteron current for 75 minutes. ^18^F2 was bubbled through a solution of 22 mg precursor in 7 mL TFA at room temperature. After delivery, TFA was evaporated at 60°C under vacuum. An additional evaporation with methanol was performed to ensure removal of TFA. The residue was dissolved in a solution of 0.2 M NaOH in 20% ethanol. The crude reaction mixture was separated on an analytical Phenomenex Gemini C18 (4.6 mm x 250 mm) column (Phenomenex, Torrance, CA) with 10% ethanol in 10 mM NaH2PO4 at a flow rate of 1mL/min. The UV detector was set at 280 nm, and the product peak was collected in a sterile vial through a sterile filter. The product peak was collected at an elution time of 30 to 43 minutes. The complete synthesis protocol is summarized in S1 Fig. Quality control procedures are described in S1 File.

### *In vivo* imaging

Subjects were administered 7.5 MBq (200 μCi) of ^18^F-EF5 by tail vein injection. In mice undergoing a terminal imaging procedure, the radiotracer was co-administered with 2.5 μmol (25 mM) of cold EF5. The total injection volume was 250 μl. The circulation time between radiotracer injection and microPET imaging was 3 hours, during which time the subjects were not anesthetized and allowed to move freely. Following anaesthetization with 2% isoflurane in oxygen at a flow rate of 0.5 L/min, subjects were imaged with an Inveon microPET/CT system (Siemens Medical Solutions USA Inc., Knoxville, TN) using a custom built 4-mouse holder. CT data were acquired as 120 projections over 360° using an 80 kVp, 500 μA beam, and were reconstructed using a filtered backprojection algorithm to yield images of size 480 x 480 x 632 voxels with an isotropic resolution of 206 μm/voxel. A static 10 minute duration PET scan was conducted immediately after the microCT scan. PET data were reconstructed using an iterative ordered subsets expectation maximization (OSEM) algorithm to yield images of dimensions 128 x 128 x 159 with a final voxel size of 776 x 776 x 796 μm. Corrections for dead time, scatter, decay, attenuation using the microCT scan, and normalization were applied to all microPET data. MicroPET images were quantified in units of percentage of injected dose per gram (%ID/g) and displayed and analyzed using the RT_Image software package [12]. Ellipsoidal regions-of-interest (ROIs) were drawn over the subscapular muscle of each mouse to quantify background muscle uptake. Tumor uptake was calculated in units of percent injected dose per gram (%ID/g) and also using the ratio of tumor to muscle uptake (T/M). Tumor ROIs were drawn manually using two methods, over the CT-visible tumor outlines (%ID/gCT and T/MCT) and over regions of enhanced ^18^F-EF5 uptake that were observable with the images windowed to 0.05–3.0%ID/g (%ID/gPET and T/MPET). For both ROI types, the mean and standard deviation of the voxels within the ROIs were measured.

### Immunohistochemistry (IHC)

After a terminal imaging examination, mice were humanely euthanized and the subcutaneous tumors were excised, fixed in formalin, embedded in paraffin, and cut into 10 μm sections. After mounting on slides, these sections were stained for EF5 using an ELK–351 anti-EF5 antibody (provided by Cameron Koch, University of Pennsylvania). Full details of the immunohistochemical staining procedure are given in the Supporting Information.

### Radiation treatment and response assessment

Subcutaneous tumors were irradiated with either a single dose (1 x 10 Gy, 1 x 40 Gy) or a daily fractionated dose (2 x 5 Gy, 2 x 10 Gy, 4 x 5 Gy, 4 x 10 Gy) using an RT–250 single-field irradiator (Philips) one day after microPET imaging. 5 mice with two tumors each were treated with each fractionation scheme, and 5 control tumor-bearing mice were not treated with radiation. Longitudinal ^18^F-EF5 imaging was performed on a separate set of mice (n = 6) with tumors treated to 0 or 20 Gy and imaged at 1 day before irradiation, and at 13 and 32 days post-irradiation.

For irradiation, mice were anesthetized and placed under a 2 mm thick lead shield with a 2 cm diameter hole placed over the tumor to restrict irradiation to the lesion. Each tumor was treated independently to a dose of 0–40 Gy based on one of the fractionation schemes. This radiation delivery strategy has been previously shown to deliver doses to subcutaneous tumors with an accuracy of ±5% [13]. Following irradiation, tumor growth was followed with sliding scale calipers for one month. Tumor volume was calculated from caliper measurements using the formula *xy^2^/2*, where *x* is the longest diameter along the surface, and *y* is the perpendicular axis [14]. Normalized tumor volumes were calculated by dividing post-treatment volumes by the pre-treatment volume. Tumor doubling times (DT) were estimated by fitting the exponential growth curve equation below to the tumor volume time series data:

$$y=y_{0}e^{\frac{t(ln2)}{DT}}$$

Tumors were classified as EF5 positive (EF5 +) or EF5 negative (EF5 -) by stratifying their T/M values based on the criteria used in previous studies [15,16], with tumors with a T/MCT value higher than 1.5 being designated as EF5 positive. Root mean square deviations (RMSD) were calculated between the EF5 positive and EF5 negative tumor volume curves as a measure of overlap, with an RMSD of zero indicating complete overlap.

### Statistical analysis

Statistical analysis of differences between EF5 positive and EF5 negative tumors was performed using a Student t-test at each time point calculated using GraphPad Prism 6.0b (GraphPad Software, La Jolla, CA).

## Results

### Radiosynthesis

The 75 minute cyclotron bombardment resulted in 11.8–12.2 GBq (319–330 mCi) at end- of-bombardment (EOB). The activity trapped in the reactor of the TracerLab system at the end of the delivery was 6.6–7.6 GBq (178–205 mCi). Upon complete evaporation of TFA the activity remaining in the reactor was 3.5–4.4 GBq (95–119 mCi). Sodium hydroxide was used to neutralize any residual TFA and to keep the final product in free form. The final dose activity ranged between 518–740 MBq (14–20 mCi) at a concentration of 37 MBq/mL (1 mCi/mL) or higher, thus enabling imaging doses to be administered intravenously in an injection volume suitable for small animals. The complete quality control analysis is documented in the Supporting Information.

### ^18^F-EF5 PET of hypoxia in subcutaneous tumor models

**Fig 1:**
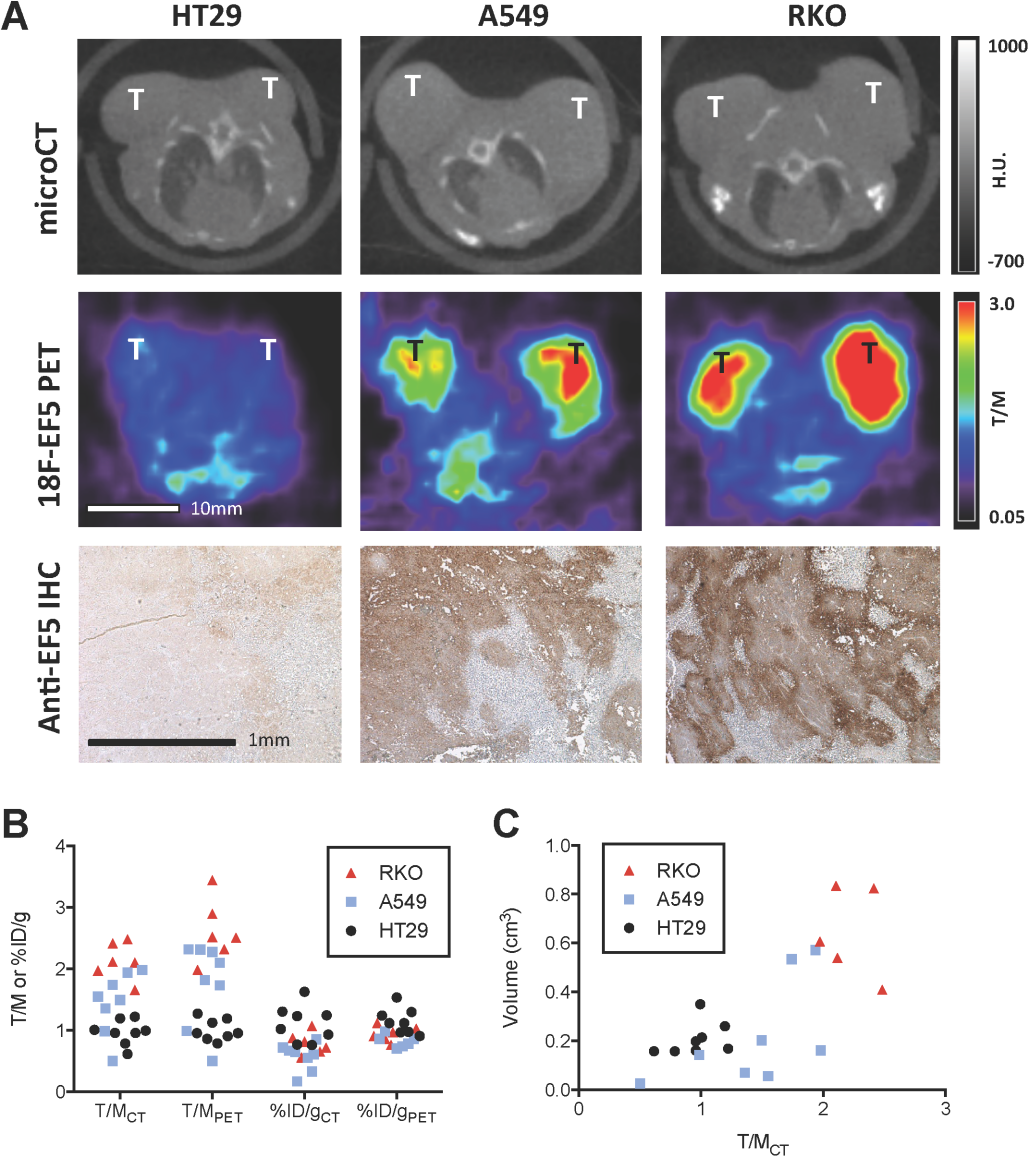
(A) Representative axial slices from microCT (top row), ^18^F-EF5 microPET (middle row), and anti-EF5 immunohistochemistry on (bottom row) of HT29, A549 and RKO subcutaneous tumors. CT image intensities represent Hounsfield Units (HU). PET images have been normalized by the mean background muscle %ID/g to yield tumor/muscle ratio (T/M) images. T on microCT and microPET images denotes tumor location. Brown stained areas in IHC images denote regions of bound EF5. (B) Scatterplot of the distribution of ^18^F-EF5 uptake in individual HT29, A549 and RKO subcutaneous tumors using three different metrics, T/M measured using CT-derived ROIs (T/MCT), T/M measured using PET-derived ROIs (T/MPET) and %ID/g values measured using CT-derived ROIs (%ID/gCT). (C) Scatterplot of the relationship between T/MCT for HT29, A549 and RKO tumors and their volume.

A total of 12 mice, 4 with subcutaneous HT29 tumors, 4 with subcutaneous A549 tumors, and 4 with subcutaneous RKO tumors, were imaged with ^18^F-EF5 microPET/CT. PET imaging examinations were performed using late stage tumors 4 weeks post-implantation. Representative results of microCT, ^18^F-EF5 microPET, and anti-EF5 immunohistochemistry studies are shown in Fig 1A. Subcutaneous tumors are clearly visible on the microCT scans. On the microPET images, the HT29 tumors were indistinguishable from the background signal, while A549 and RKO tumors were clearly visible, with RKO tumors exhibiting the greatest uptake. Anti-EF5 staining of these tumor specimens following animal sacrifice and tissue harvesting was in agreement with the *in vivo* findings, with 5 ± 3% of tumor regions staining positive in the HT29 tumors, 52 ± 14% positive in the A549 tumors, and 71 ± 19% positive in the RKO tumors. The tumor uptake values from this subject population are summarized in Fig 1B. HT29 tumors exhibited T/MCT and T/MPET values of approximately 1, indicating no significant uptake beyond background levels. T/MCT values for RKO tumors were approximately 2, while T/MPET values were significantly greater than 2 (mean 2.6±0.5). A549 tumors demonstrated a broad range of uptakes between these two extremes (mean T/MPET 1.8±0.7, mean T/MCT 1.4±0.5). The T/M values corresponded with tumor EF5 levels in the *in vivo* and IHC images. In contrast, the %ID/g values for each tumor showed an inverse correlation, with HT29 tumors exhibiting higher %ID/gCT and %ID/gPET values than RKO and A549 tumors. This was due to higher background ^18^F-EF5 uptake in the HT29 tumor mice, which was accounted for by the T/M measurements. Fig 1C shows the relationship between ^18^F-EF5 uptake and tumor volume. HT29 and A549 tumors were of similar sizes (mean volume 0.21±0.07 cm3 and 0.22±0.21 cm3 respectively), while RKO tumors were significantly larger (mean volume 0.79±0.39 cm3). The heterogeneity of EF5 uptake observed in the A549 tumors led us to utilize this model for the subsequent experiments to investigate the relationship between pre-treatment EF5 uptake and radiation response.

### Radiotherapy response

**Fig 2:**
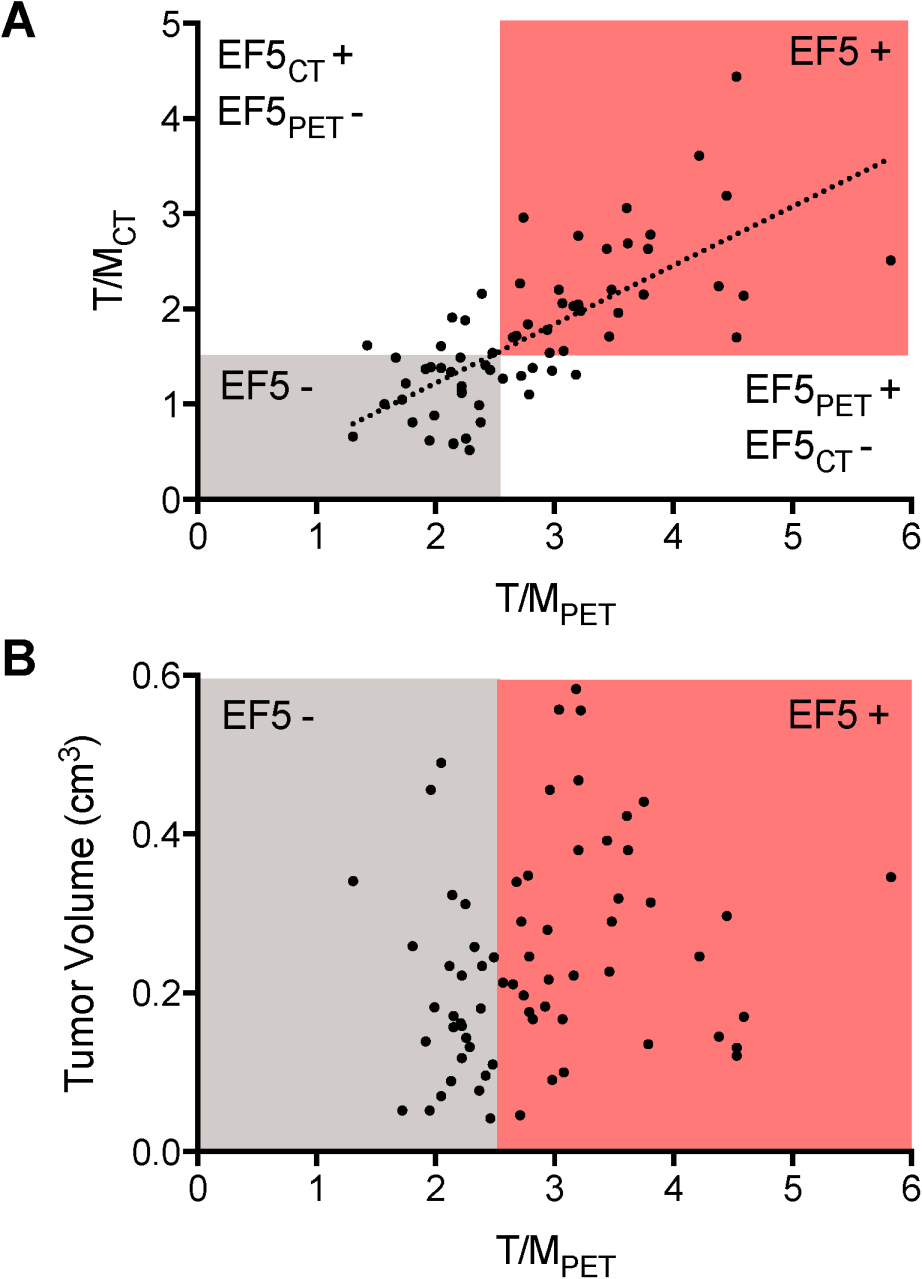
(A) Scatterplot of the relationship between ^18^F-EF5 T/M values for individual tumors when quantified using either PET and CT derived ROIs (T/MPET and T/MCT respectively), with a linear regression line superimposed. The four quadrants represent regions where both T/MPET and T/MCT are below the designated hypoxia threshold (EF5 -), or both are above the threshold (EF5 +), or only one is above the threshold (EF5CT+/EF5PET- or EF5CT-/EF5PET+). (B) Scatterplot of ^18^F-EF5 tumor uptake vs tumor volume. The gray region denotes EF5 negative tumors (defined as T/MPET < 2.5), and the red region denotes EF5 positive tumors (T/MPET > 2.5).

In a second experiment, a total of 40 mice, each bearing two subcutaneous A549 tumors, were treated with a variety of radiotherapy protocols. Tumors were grown for six weeks, after which all mice were imaged with ^18^F-EF5 microPET/CT and classified as EF5 positive or EF5 negative. Fig 2A shows a scatterplot of individual A549 T/MPET values vs T/MCT values. A linear correlation was observed between measurements obtained using the two metrics (r2 = 0.51). The equation for the regression line, *T/MCT = 0*.*61 T/MPET*, yields a threshold T/MPET value of 2.46 as equivalent to the threshold T/MCT value of 1.5. A total of 13 tumors were designated as EF5 positive according to one T/M value (T/MPET or T/MCT), but EF5 negative according to the other. The values in the EF5CT +/EF5PET–box in Fig 2A reflect the uncertainty involved in quantifying tumor ^18^F-EF5 uptake due to misalignments of the PET and CT images and potential spillover of physiological signal into the CT-based tumor ROI, while values in the EF5CT—/EF5PET + box are likely due to the EF5 hotspot being small relative to the size of the tumor. The latter category is still considered to be a hypoxic tumor, and therefore the T/MPET threshold value is used for all subsequent analysis.

Fig 2B shows a scatterplot of individual A549 T/MPET values against volume, classified by hypoxic status using a T/MPET of 2.5. The threshold divides the population into 39 EF5 positive and 41 EF5 negative tumors. Both groups contain a large spread of volumes, and the volumes are higher for the EF5 positive group (mean EF5 positive volume 278±135 mm3, mean EF5 negative volume 190±114 mm3, p<0.01). However there is no linear correlation between volume and ^18^F-EF5 uptake when treated as a continuous variable (r2 = 0.04).

**Fig 3:**
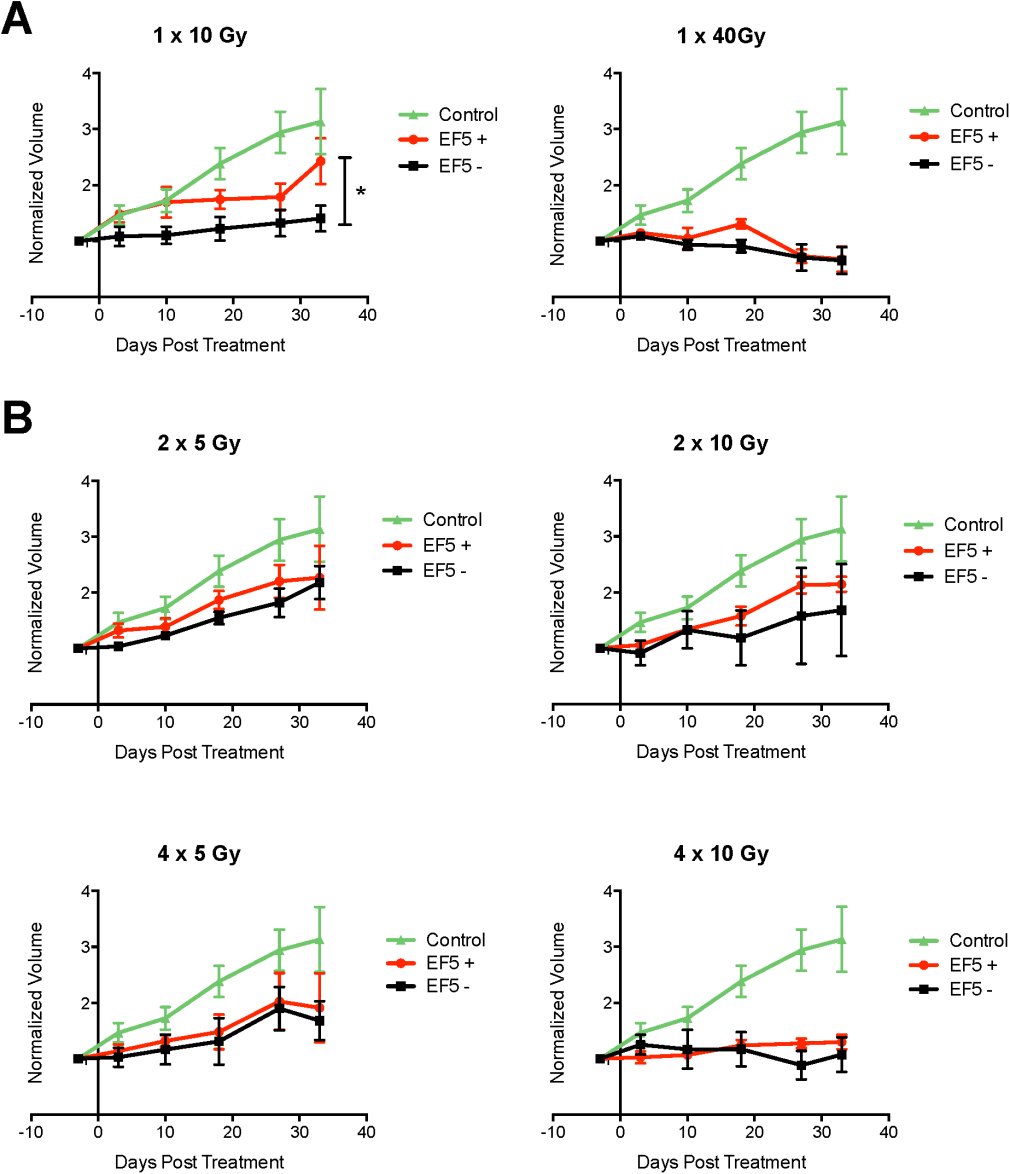
Plots of response of more and less hypoxic tumors to (A) single fraction and (B) multiple fraction treatments. Volumes are normalized to pre-treatment volumes.

Following imaging, tumors were irradiated and monitored with caliper measurements, and the volumes were normalized by pre-treatment volumes. The tumor growth profiles are shown in Fig 3A (single doses) and B (fractionated doses). DT and RMSD values are summarized in Table 1. Pre-treatment tumor DT values are 9.6 days for EF5 positive tumors and 10.1 days for EF5 negative tumors. Post-treatment, the control (unirradiated) tumors tripled in volume after one month. In the 1 x 10 Gy group, the EF5 positive and EF5 negative growth curves are well resolved (P < 0.01; RMSD of 0.64), and DT values were 39 days (EF5 positive) and 75 days (EF5 negative). The 1 x 40 Gy group responded very differently, with the EF5 positive and EF5 negative tumor time courses showing more overlap (RMSD of 0.19), and both resulting in a 35% decrease in tumor volume.

**Table 1:** Doubling Times (DT) and Root Mean Squared Deviation (RMSD) between EF5 positive and EF5 negative tumor time curves for pre-treatment and post-treatment A549 subcutaneous tumors. Values of n/a are reported for growth curves that were flat or decreasing over time.

Treatment	Number of Tumors		DT		RMSD
Group	EF5+	EF5-	EF5+	EF5-	
Pre-Treatment	36	37	9.6 ± 1.1	10.1 ± 1.5	n/a
Control (0 Gy)	0	7	22.6 ± 3.9	24.9 ± 4.6	n/a
1 x 10 Gy	4	6	38.8 ± 10.0	74.9 ± 38.0	0.64
1 x 40 Gy	3	6	n/a	n/a	n/a
2 x 5 Gy	3	6	32.6 ± 7.7	30.1 ± 4.8	0.27
2 x 10 Gy	6	3	30.2 ± 3.2	43.3 ± 33.2	0.37
4 x 5 Gy	6	4	36.1 ± 14.2	38.6 ± 14.7	0.16
4 x 10 Gy	6	4	86.3 ± 25.5	n/a	0.23

The 2 x 5 Gy group shows a reduced separation between EF5 positive and EF5 negative groups, and a poorer overall response compared to the 1 x 10 Gy group, with DT values of 33 days (EF5 positive) and 30 days (EF5 negative). The 2 x 10 Gy and 4 x 5 Gy groups show similar levels of tumor control, with DT values of 30–36 (EF5 positive) and 39–43 (EF5 negative), but the 4x fractionation regimen shows an increased overlap between EF5 positive and EF5 negative tumor time courses (RMSD 0.16 for 4 x 5 Gy versus 0.37 for 2 x 10 Gy). Finally, the 4 x 10 Gy group shows a high level of overlap (RMSD 0.23), but a lower level of tumor control compared to the 1 x 40 Gy group, with the tumor volumes staying constant throughout the observation period.

**Fig 4:**
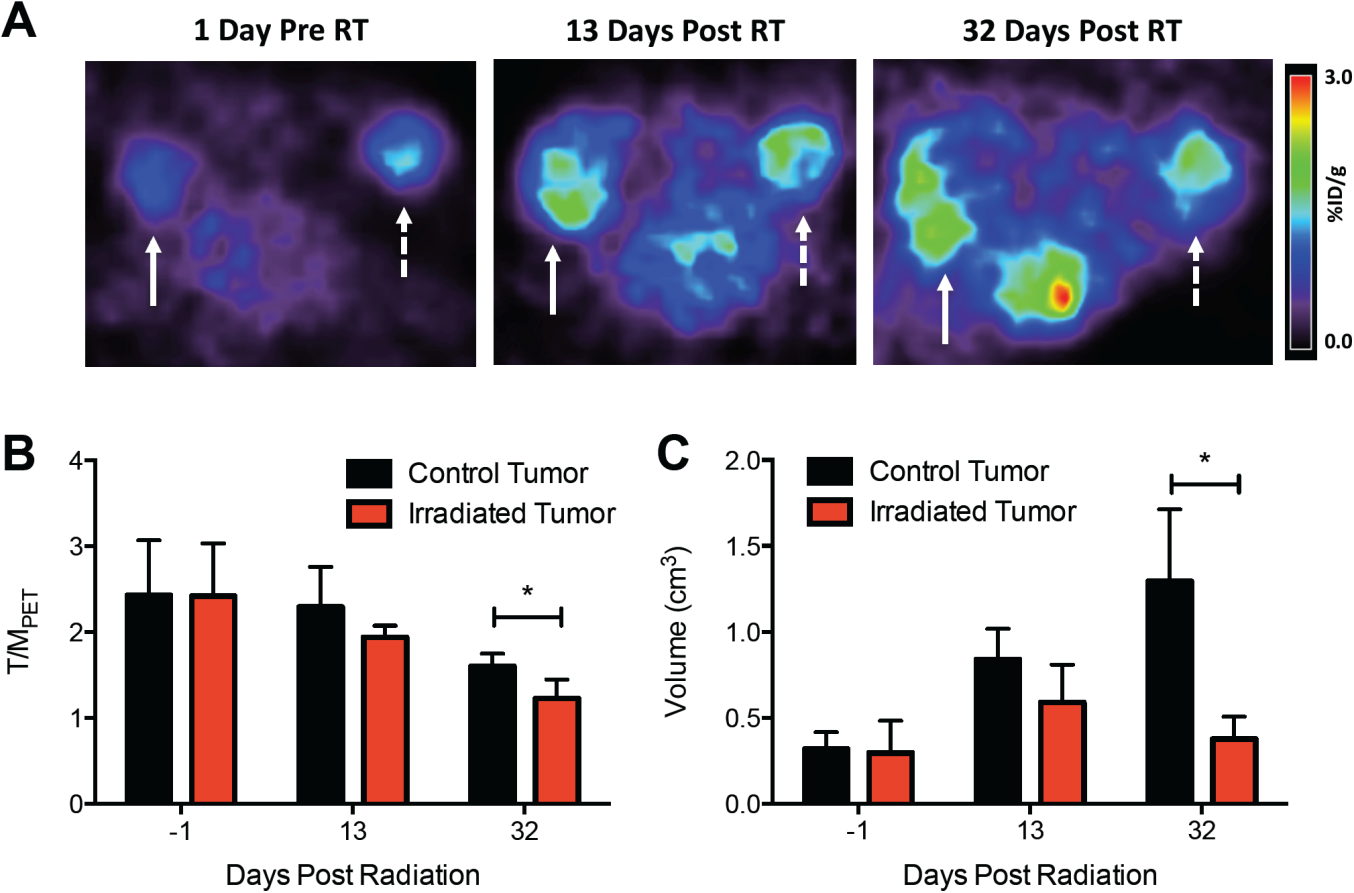
Effect of radiation on tumor ^18^F-EF5 uptake. (A) Representative axial ^18^F-EF5 PET images of two tumors at 1 day pre-treatment and 13 / 32 days post-treatment. Solid arrow denotes unirradiated control tumor, and dashed arrow denotes tumor which was treated with 20 Gy. (B,C) Plots showing changes in ^18^F-EF5 uptake and volume, respectively, in control (n = 6) and irradiated (n = 6) tumors during the treatment timecourse.

Fig 4A shows the changes in tumor hypoxia post-irradiation as revealed by ^18^F-EF5 microPET imaging. The control tumor increased in volume and ^18^F-EF5 uptake over the course of a month, whereas the irradiated tumor shows a smaller increase in size and ^18^F- EF5 uptake. Fig 4B compares the T/MPET values for the control and irradiated tumors at each time point. Prior to treatment, there is no difference between the two groups. After 13 days, there is a reduction in the T/MPET of the irradiated group that is not statistically significant. After 32 days, there is a noticeable decrease in the T/MPET for both groups, and the irradiated tumor T/MPET is significantly lower than that of the control group (p = 0.03). Fig 4C shows an increase in tumor volume for control tumors, while the irradiated tumor volume remains constant over 32 days. H&E sections of tumors after 32 days reveal large regions of necrosis in control tumors, and smaller necrotic regions accompanied by macroscopic blood pools in treated tumors (S4 Fig).

## Discussion

The aim of this study was to evaluate the ability of pre-treatment ^18^F-EF5 PET imaging to predict the efficacy of radiation therapy. ^18^F-EF5 has received considerable attention recently as it has the potential to serve as an clinically-translatable hypoxia-specific PET tracer for imaging hypoxia. Its pharmacokinetic profile compares favorably with that of ^18^F-FMISO, and it has the added advantage of being directly comparable to microscopic hypoxia through the use of established anti-EF5 antibodies for immunohistochemistry [17].

Subcutaneous tumors are known to be strongly hypoxic [18], and we surveyed several tumor models to identify one that produced both EF5 positive and EF5 negative tumors. Subcutaneous A549 and RKO tumors have previously been shown to exhibit strong uptake of ^18^F-FAZA [18,19], whereas HT29 subcutaneous tumors have been reported to take up moderate levels of ^18^F-FMISO [20]. Fig 1 shows *in vivo* and immunohistochemical images of EF5 uptake in HT29, A549 and RKO tumors. Overall uptake values for all three tumor types was low, with %ID/gCT values ranging between 0.33–1.63. A549 tumors were found to generate the broadest spread of *in vivo* ^18^F-EF5 uptake, with a T/MCT 95% confidence interval of 1.03–1.86. Previous studies have proposed the use of a T/M cut-off value of 1.5 as a threshold for designating tumors as hypoxic [15,16] when imaging with ^18^F-EF5 or ^18^F-FAZA PET. Under this criteria, our pilot study yielded 4 EF5 positive and 4 EF5 negative A549 tumors, suggesting that it would be a good model in which to study hypoxia-dependent radiation response in a genetically matched tumor system. The ^18^F-EF5 uptake levels observed in tumors in this study agreed quantitatively with those reported in previous studies [8,11,15].

Hypoxia is commonly modeled as a function of volume, as tumors that grow too large outstrip their oxygenation and nutrient supply due to the inability of nutrient supply by the tumor vasculature to keep up with increasing metabolic demand. The scatterplot in Fig 2B shows that when tumors are stratified into EF5 positive and EF5 negative groups, there is a significant difference in tumor volume, however when ^18^F-EF5 uptake is treated as a continuous variable, there is no correlation. This suggests that volume alone is insufficient to explain the observed variability in A549 tumor ^18^F-EF5 uptake, and that other factors are involved. One possibility is that stochastic differences in microvascular structure and function between tumors contributes to the observed variation. Clinically, this observation suggests that hypoxia as measured by PET imaging is not simply a surrogate for tumor volume.

Our radiotherapy results show that when tumors are stratified on the basis of their pre- treatment ^18^F-EF5 uptake into EF5 positive and EF5 negative groups, different patterns of response are observed for each group following single and multiple fraction treatments. Previously, ^18^F-EF5 uptake in rat 9L gliomas prior to radiation treatment has been associated with the subsequent *in vitro* survival of these cells [8]. In addition, ^18^F-EF5 was shown to predict the outcome of combined radiation and SN30000 treatment in rats bearing human A460 non-small cell lung cancer xenografts [11]. In the present study we examined this relationship across a variety of radiation fractionation schemes, and observed that ^18^F-EF5 was predictive of treatment outcome for single fraction treatments, but that addition of fractions increased the overlap between EF5 positive and EF5 negative tumor response curves. EF5 positive tumors are noticeably more resistant to a treatment of 1 x 10 Gy than EF5 negative tumors, whereas after 1 x 40 Gy the response curves for these two groups of tumors overlap, suggesting higher doses can overcome the inherent resistance in hypoxic tumors. However this dose level is highly ablative and is at the limit of what is clinically practical. Increasing the number of fractions results in a narrowing of the responses between EF5 positive and EF5 negative tumors, with treatments using four fractions resulting in converging responses. Overall, these suggest that pre-treatment ^18^F-EF5 PET can be utilized to predict the influence of hypoxia on expected tumor control after irradiation with a single or multiple fraction treatment,. They also suggest that an *a priori* knowledge of the tumor hypoxic status can determine the optimal treatment. For EF5 negative tumors, smaller fractions have a stronger tumor control effect, as 1 x 10 Gy results in DTs of 75 days, while 2 x 5 Gy results in DTs of 30 days. However for EF5 positive tumors, larger fractions obtain outcomes that are almost as potent.

The post-irradiation tumor images show that radiotherapy arrests the development of the EF5-trapping phenotype, with irradiated tumors displaying significant decreases in both ^18^F-EF5 uptake and volume relative to the control tumors at one month post-treatment, but not after two weeks. A previous study examined subcutaneous tumor uptake of ^18^F- FAZA immediately after fractionated radiotherapy and did not find a significant decrease in uptake. Combined with our results, this suggests that the effects of reoxygenation become apparent by PET imaging several weeks after treatment [21]. H&E stained sections acquired after the final imaging time point show severe necrosis in the control tumors, but minimal necrosis in the treated tumors (data not shown). It is unclear why the control tumor uptake also decreases in our data, however the presence of significant necrosis could indicate a reduction in the proportion of viable hypoxic cells that can trap ^18^F-EF5 in these late-stage tumors.

There are several limitations to our study. The use of subcutaneous tumors is of limited applicability to clinical tumors that are typically more genetically heterogeneous. However we chose this model in order to employ a well-defined and genetically homogeneous model in which to observe the effects of hypoxia and the ability of hypoxia imaging to detect these effects. In addition, our radiotherapy study used large tumors with a minimum diameter of 8 mm. The largest dose studied, 1 x 40 Gy, resulted in a tumor volume reduction of 35%, and this could be because the tumors comprised significant regions of necrosis that are unaffected by radiation. Finally, our study follows the accepted approach of stratifying tumors into two distinct groups based on their level of hypoxic tracer uptake. This simplifies the effect of cellular oxygen tension, whereas recent radiobiological research has shown that oxygenation should ideally be treated as a continuous spectrum [22]. Investigating the predictive ability of hypoxia-specific PET tracer uptake as a continuous variable will require a more complex study with significantly larger group sizes, potentially combined with careful use of hypoxia modifying agents to manipulate tumor hypoxia levels. The question of hypoxia as a continuous variable raises the issue of how to best infer this value from hypoxia imaging. Further consideration of the most appropriate quantitative methods to analyze hypoxia PET images is warranted.

Our study is particularly relevant to the development of hypoxic lung tumor treatments using hypofractionated protocols. Interest in hypofractionation has increased recently due to the ability to conform the radiation dose more closely to the tumor volume using Stereotactic Body Radiotherapy (SBRT) [23], however this approach is more vulnerable to hypoxia due to the loss of the reoxygenation effect that accompanies fractionation. Identifying hypoxic tumors prior to hypofractionated radiotherapy would allow the hypoxic resistance effects to be directly addressed, either by an oxygen modulating treatment such as carbogen or by the use of hypoxia-specific radiosensitizers. Our results suggest that ^18^F-EF5 PET offers a clinically translatable form of hypoxia imaging that can be used to inform the planning of current radiation treatment protocols.

## Supporting Information

S1 Fig Schematic diagram for the preparation of ^18^F-EF5 using the GE FX-FE module. (PDF)

S2 Fig (A) HPLC chromatogram for attempted purification of ^18^F-EF5 on a semiprep column. Conditions: Phenomenex Gemini C18 (10 x 250 mm), 10% or 9% ethanol in 10mM NaH_2_PO_4_, 5.0 mL/min, λ = 280 nM. (B) HPLC chromatogram for purification of ^18^F-EF5. Synthesis conditions: Phenomenex Gemini C18 (4.6 x 250 mm), 0% ethanol in 10mM NaH_2_PO_4_, 1.0 mL/min, λ = 280 nM. The broad peak from 32 min to 47 min was collected as the product.
(PDF)

S3 Fig (A) HPLC chromatograms of crude EF5 product by a Phenomenex Gemini C18 (4.6 x 250 mm) column, 50%/50% MeCN/water, isocratic gradient, 1.0 mL/min, λ = 325 nM. (B) HPLC chromatograms for quality analysis of ^18^F-EF5: Phenomenex Gemini C18 (4.6 x 250 mm), 50%/50% MeCN/water, isocratic gradient, 1.0 mL/min, λ = 325 nM. Radiochemical purity >98%. (C) HPLC chromatograms to confirming the purity of ^18^F-EF5 on different stationary phase: Phenomenex Gemini C6-phenyl (4.6 x 250 mm), 50%/50% MeCN/water, isocratic gradient, 1.0 mL/min, λ = 325 nM. Radiochemical purity >98%. The top plot in each figure shows the output in the radioactive channel (measuring activity of elutants), and the bottom plot shows the output in the UV channel (measuring absorbance at 325 nm).
(PDF)

S4 Fig H&E sections of a control and irradiated tumor at 32 days post-treatment. (PDF)

S1 File Detailed description of the radiosynthesis and quality control of ^18^F-EF5, and a detailed summary of the harvesting and immunohistochemical analysis of tissues from tumor-bearing mice. (DOCX)
