# Supplementary figures and images for: 18F-EF5 PET Is Predictive of Response to Fractionated Radiotherapy in Preclinical Tumor Models
Source: PLoS One. 2015 Oct 2;10(10):e0139425. doi: 10.1371/journal.pone.0139425 (PMC4592127; doi:10.1371/journal.pone.0139425)

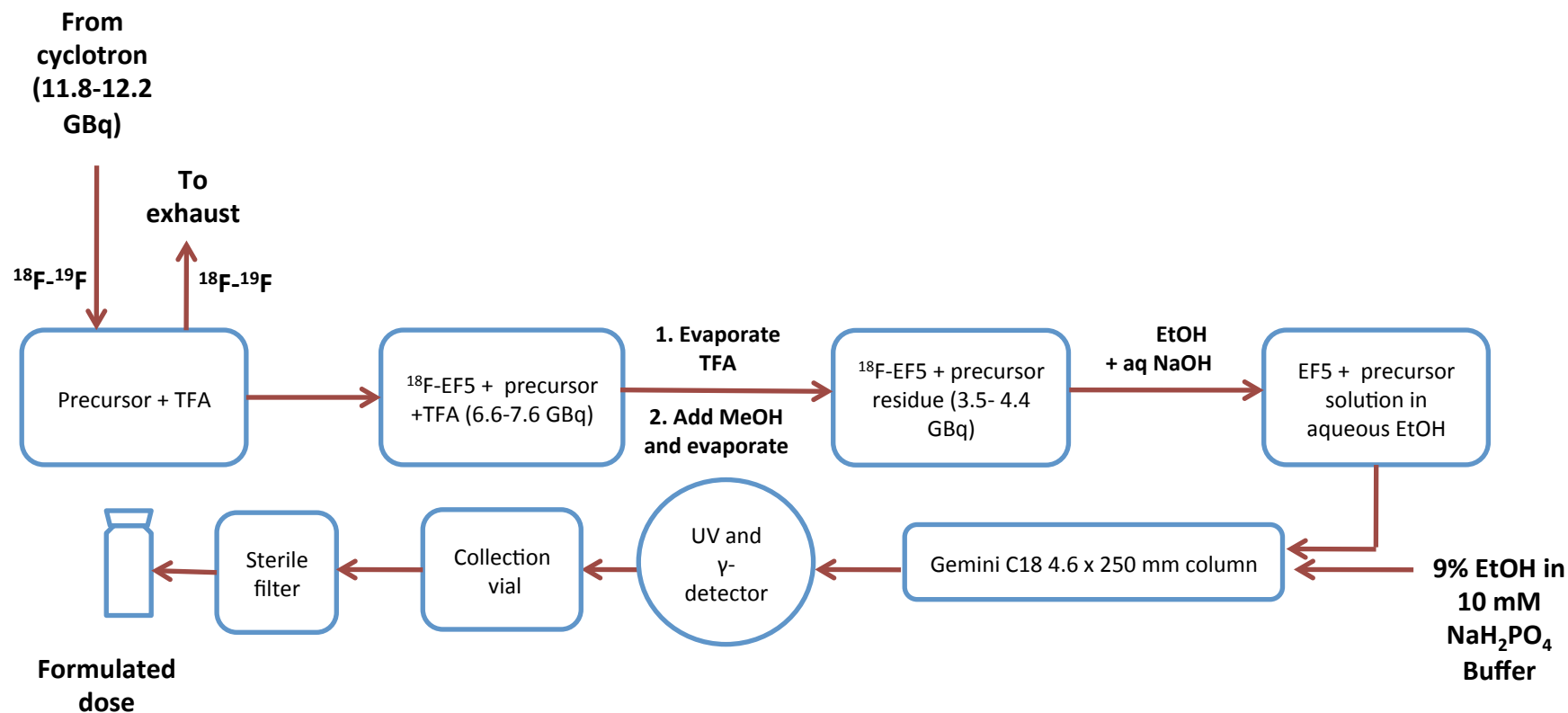

**Figure S1**

Supplement: S1 Fig — (PDF) [file pone.0139425.s001.pdf]

**A**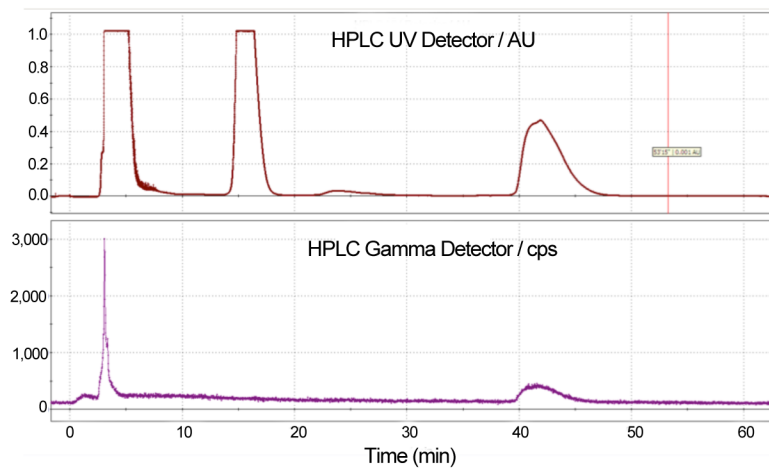**B**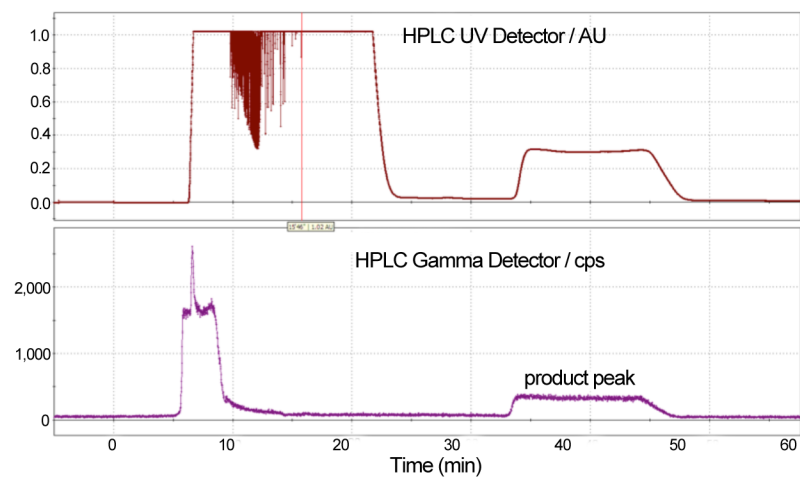**Figure S2**

Supplement: S2 Fig — The broad peak from 32 min to 47 min was collected as the product. (PDF) [file pone.0139425.s002.pdf]

**A**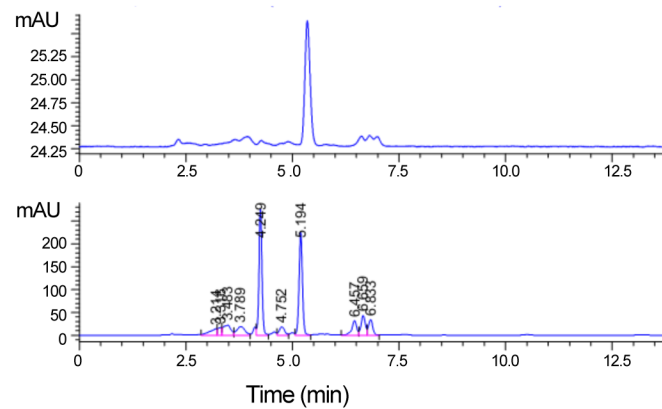**B**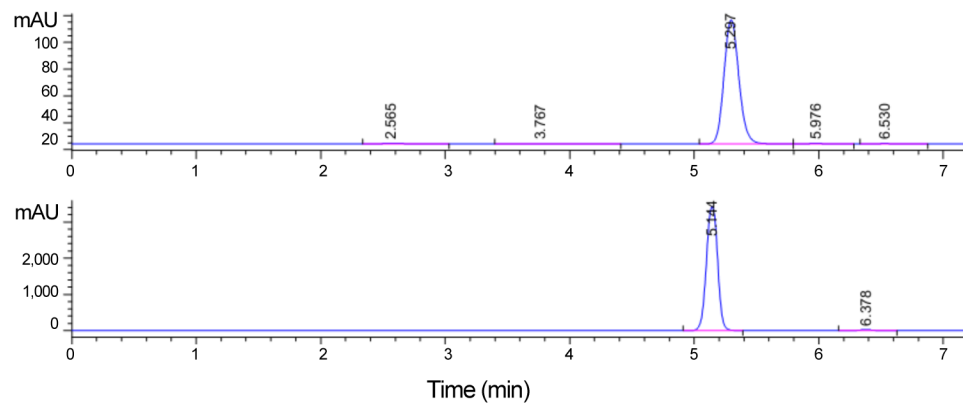**C**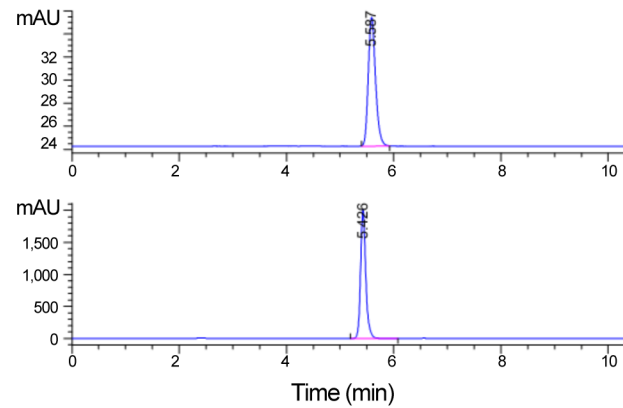**Figure S3**

Supplement: S3 Fig — The top plot in each figure shows the output in the radioactive channel (measuring activity of elutants), and the bottom plot shows the output in the UV channel (measuring absorbance at 325 nm). (PDF) [file pone.0139425.s003.pdf]

**Control**

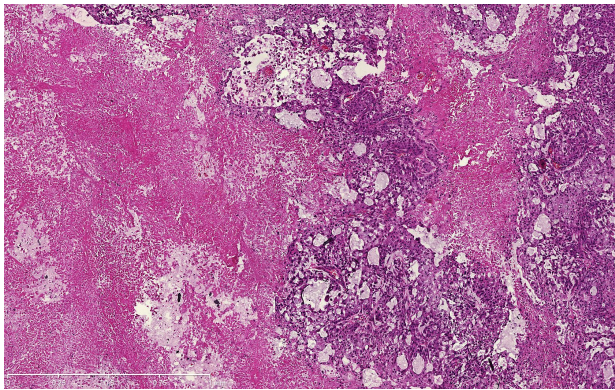

**Irradiated**

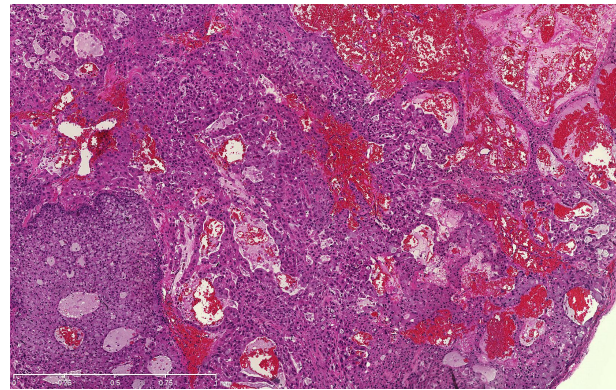

1mm

**Figure S4**

Supplement: S4 Fig — (PDF) [file pone.0139425.s004.pdf]
