# Supplementary material for: 18F-EF5 PET Is Predictive of Response to Fractionated Radiotherapy in Preclinical Tumor Models
Source: PLoS One. 2015 Oct 2;10(10):e0139425. doi: 10.1371/journal.pone.0139425 (PMC4592127; doi:10.1371/journal.pone.0139425)
Supplement: S1 File — (DOCX) [file pone.0139425.s005.docx]

**Additional Materials and Methods**

18F-EF5 Quality Control

For Quality Control (QC), a 1.2 mL aliquot was removed from the final dose vial using a pre-inserted syringe. Quality assurance measurements were carried out on an Agilent 1100 series HPLC system (Agilent, Santa Clara, CA) with a Gabistar radiodetector using a Phenomenex Gemini C18 HPLC column (4.6 mm x 250 mm), with a mobile phase consisting of water and acetonitrile (1:1 ratio) at a flow rate of 1.0 mL/min. The UV detection wavelength was 325 nm and the retention time for the EF5 peak was 5.1 minutes. The specific activity was determined from a standard curve, which was obtained from the UV peak areas observed for injections of different concentrations of the cold EF5 standard. The identity of the product was confirmed by coinjection with a cold EF5 sample, and comparison of peaks in the radiation and UV detector channels.

Analysis of residual organic solvents in the product was carried out using an Agilent 6850 GC (Agilent Technologies Inc., USA). Apyrogenicity (Charles River Laboratories Inc., USA) tests were performed to evaluate endotoxin levels in the final dose. The integrity of the sterile filter was tested by a bubble point test at 45 psi. Sterility tests were performed using standard protocols.

Immunohistochemistry

Following completion of imaging, mice were sacrificed by CO_2_ asphyxiation and cervical dislocation. Tumors were excised and fixed in formalin in a cold room. After 24 hours, the formalin was replaced with PBS, and the samples were wax-embedded and sectioned. Tissue sections were dewaxed by washing in xylene, followed by rehydration by immersion in 100%, 95%, 85%, 70% ethanol and demineralised water (5 minutes per wash). Antigen retrieval was performed by incubating slides in 10 mM citric acid (pH 6.0) for 15 minutes in a 700W microwave at full power. Non-specific binding sites were blocked by washing with Avidin D and Biotin solutions (Vector Labs, Burlingame, CA), and 10% mouse serum (KPL, Gaithersburg, MD). Slides were incubated overnight on a rocker at -4^o^C with regular and competed stain versions of the ELK351-Biotin antibody (provided by Cameron Koch, University of Pennsylvania) or with 0.1% Tween/PBS solution for the negative control. After washing, the slides were incubated with avidin/streptavidin antibody conjugated to horseradish peroxidase (HRP). Staining was developed by washing with DAB+ chromogen (Dako, Carpinteria, CA) until brown coloration became visible. After final washing, slides were fixed with Fluromount G (SouthernBiotech, Birmingham, AL), and imaged using a Leica CTR 6000 microscope with a Leica DFC color camera (Leica Microsystems GmbH, Wetzlar, Germany) at 5x magnification. One slice per tumor was separately stained with hematoxylin and eosin (H&E).

**Additional Results**

18F-EF5 Synthesis

Figure S1 summarises the yields obtained after each step. In a previously reported EF5 synthesis protocol using solid phase extraction (SPE) (1), it was found that approximately 40% of the product is lost during the extraction. Our approach involves elution of the HPLC column with a formulated mobile phase containing a maximum of 10% ethanol, eliminating SPE. Such a limitation on the mobile phase and the highly hydrophobic nature of EF5 made it necessary to use an HPLC column that offered the least retention in order to elute the product out in a practical timeframe and to minimize the final dose volume in order to obtain high concentration products. A preliminary purification experiment was performed using a C-18 semi-prep column (10mm x 250mm). This eluted the product in 40 minutes with a flow rate of 5.0 mL/min, however the dose volume was 25-30 mL (Figure S2A), resulting in a final product which was too dilute for imaging applications. A C-18 analytical column (4.6mm x 250mm) was then tested. This eluted the product in 50 minutes in a dose volume of 12-17 mL yielding a product which was approx 1 mCi/mL which is suitable for performing tail-vein injections where injection volumes must be kept below 250 µl. This column is stable at basic pH, including the pH of the injected sample prior to neutralization by the mobile phase. The product exhibited a broad, plateaued peak of width 12-17 minutes, which was clearly separated from the precursor peak (Figure S2B). The final dose activity ranged between 518-740 MBq.

An aliquot of crude reaction mixture from the reactor was injected on the C-18 analytical column to assess its ability to separate the product from precursor and other impurities (Figure S3A). The purity of the product was determined using analytical HPLC. The radiochemical purity was >98% and the chemical purity was >97% (Figure S3B). In order to confirm that the product peak did not contain underlying impurity peaks, the experiment was repeated with a Phenomenex Gemini C6-Phenyl HPLC column (4.6 mm x 250 mm) (Figure S3C) under the same conditions as for the C-18 analytical column, and the purity of the resulting sample matched that from the C-18 analytical column. The specific activity ranged from 66 to 122 MBq/µmol. Due to the small volume of the analytical column, the amount of ethanol in the injection sample was found to be critical for good separation and reproducible retention time (RT). A higher ethanol concentration was found to change the product retention time significantly, and to merge the product peak with the precursor peak.

The column back pressure was 120 bar before the sample injection. After injection the pressure rose to 190 bar as 20% ethanol passed through the column, before dropping back to 120-125 bar after 10 minutes of elution. No significant effect on yield was found by increasing the cyclotron bombardment time.

(1) Dolbier WR, Li A-R, Koch CJ, Shiue C-Y, Kachur A V. [18F]-EF5, a marker for PET detection of hypoxia: synthesis of precursor and a new fluorination procedure. Appl Radiat Isot. 2001;54:73–80.
